# Is the Association of the Rare rs35667974 *IFIH1* Gene Polymorphism With Autoimmune Diseases a Case of RNA Epigenetics?

**DOI:** 10.1007/s00239-022-10090-0

**Published:** 2023-01-18

**Authors:** Athena Andreou, Athanasios Papakyriakou, Maria I. Zervou, George N. Goulielmos, Elias E. Eliopoulos

**Affiliations:** 1 Laboratory of Genetics, Department of Biotechnology, Agricultural University of Athens, 11855 Athens, Greece; 2 Institute of Biosciences and Applications, National Centre for Scientific Research “Demokritos”, 15341 Athens, Greece; 3 Section of Molecular Pathology and Human Genetics, Department of Internal Medicine, School of Medicine, University of Crete, 71003 Heraklion, Greece; 4 Department of Internal Medicine, University Hospital of Heraklion, 71500 Heraklion, Greece

**Keywords:** Single-nucleotide polymorphism (SNP), Molecular model, Interferon induced with helicase C domain 1 (IFIH1), Melanoma differentiation-associated 5 (MDA5), RNA methylation

## Abstract

Interferon induced with helicase C domain-containing protein 1 (IFIH1) gene encodes a cytoplasmic RNA helicase otherwise known as melanoma differentiation-associated 5 (MDA5), a RIG-1-like RNA helicase that recognizes viral RNA and is involved in innate immunity through recognition of viral RNA. Upon binding to double-stranded (ds) RNA, MDA5 forms a filamentous assembly along the length of dsRNA and utilizes molecular signatures to discriminate self, versus non-self on the basis of dsRNA length and methylation. Its missense variant rs35667974 is protective for type 1 diabetes, psoriasis, and psoriatic arthritis, but is also found to be associated with an increased risk for ankylosing spondylitis, Crohn’s disease, and ulcerative colitis. To gain insight into the complex role of this variant we performed a structural analysis of MDA5 in complex with dsRNA using molecular dynamics simulations. Our data suggest that while the Ile923Val mutation of the rs35667974 variant does not affect binding to native dsRNA significantly, it displays a destabilizing effect in the presence of 2′-O uridine methylation. Thus, the presence of 2′-O-methylation at the dsRNA introduces a sensing signature that leads to selective reduction of the overall MDA catalytic activity. This study represents an evaluation of the role of the shared rs35667974 variant of autoimmune locus IFIH1, reported to lead to selectively reduced catalytic activity of the modified MDA5 phenotype and, as a consequence, reduced negative feedback on cytokine and chemokine signaling and selectively protection against autoimmunity.

**Supplementary Information:**

The online version contains supplementary material available at 10.1007/s00239-022-10090-0.

## Introduction

Genes and mechanisms involved in autoimmune diseases, which affect approximately 5% of the population, remain still elusive but accumulating data strongly suggest that different autoimmune diseases can share a common genetic background, thus pointing out the existence of variants that are shared by different autoimmune diseases (Zhernakova et al. 2009). Attempting to unravel this genetic information into biologically meaningful mechanisms leading to diseases refers to the identification of the causal genes. Identification of disease-causing variants is a difficult but necessary task in the attempt to establish effective methods for disease prediction, prevention, and intervention (Biros et al. 2005).

There are different types of RNA molecules that are involved in the regulation of several biological processes, including messenger RNA (mRNA), transfer RNA (tRNA), ribosomal RNA (rRNA), microRNA (miRNA), and long noncoding RNA (lncRNA). RNA molecules contain numerous (more than 150) chemical modifications (Machnicka et al. 2013; Boccaletto et al. 2018). These modifications are functionally linked to all stages of RNA metabolism, such as structure, stability, and interactions, and play critical roles in several biological processes, such as modulating the replication of viruses and antiviral immune responses (Machnicka et al. 2013). Among them ribose methylation is among the most ubiquitous modifications found in RNA. 2′-O -methyluridine is found in rRNA, snRNA, snoRNA, and tRNA of Archaea, Bacteria, and Eukaryota (Aučynaitė et al. 2018). The ribose-2′-*O*-methylation increases the hydrophobicity of nucleotides and protects them against the action of nucleases (Yildirim et al. 2014).

Accumulating evidence indicate that 2′-O-methylation of viral RNA (2′OMe-RNA) plays an important role in evasion of cellular innate immune responses in the host cells (Dimitrova et al. 2019). Züst and colleagues have shown that 2′ OMe of viral RNA contributed to evasion of the interferon (IFN)-mediated antiviral response, thereby promoting viral replication (Züst et al. 2011). Also, Vitali and Scadden have proposed that IU-dsDNA suppresses the MDA5 IFNβ-stimulating pathway (Vitali and Scadden 2010).

Interferon induced with helicase C domain 1 (*IFIH1*) gene encodes a cytoplasmic RNA helicase also known as MDA5 (Melanoma differentiation-associated protein 5), and it is a RIG-I-like receptor (RLR) that performs antiviral function in innate immunity by detecting viral RNAs. MDA5 recognizes 0.5–1 kb RNA duplex stem structure that is usually formed during picornaviral replication and mediates an immune response on viral infection (Nejentsev et al. 2009; Crow 2011). MDA5, upon detection of long viral double-stranded RNAs (dsRNAs), generated during replication of picornaviruses, activates the type I interferon signaling pathway. Studies have shown that MDA5 forms a filament along the length of dsRNA and utilizes ATP-dependent filament dynamics to discriminate between self vs non-self on the basis of dsRNA length (Toro et al. 2015). MDA5 was shown to be involved in the modulation of crosstalk between β cells and the innate/adaptive immune system through the local production of cytokines and chemokines. Changes in MDA5 expression and/or activity may trigger β-cell responses to dsRNA, a by-product of virus replication (Colli et al. 2010). It has also been shown that mutation of filament forming-associated residues results in loss of filament formation and MDA5-dependent signaling, except for a pair of mutations, which moderately enhance signaling. These results suggest that ATP-independent mechanisms, i.e., tighter RNA binding and/or more stable protein–protein interaction, are likely to be responsible for the observed stability of the MDA5 filament formation in vitro and for higher signaling activity in cells (Sohn and Hur 2016).

Smyth et al. (2006) and Nejentsev et al. (2009) described a rare allele of *IFIH1* gene that confers protection from type 1 diabetes (T1D). This rs35667974 *IFIH1* single-nucleotide polymorphism (SNP), where a conserved isoleucine (codon [ATT]) at position #923 changes to valine (codon [GTT]), is a rare variant as the minor allele frequency (MAF) is *C* = 0.010031 (2655 individuals in a total sample of 264690) based on TOPMED (Taliun et al. 2021) and *C* = 0.016267 (3343 individuals in a total sample of 205514) based on ALFA (Phan et al. 2020; Sherry et al. 2001). In Table 1 the human biogeography of the frequency of the polymorphism in different continental regions based on the ALFA project data (Phan et al. 2020; Sherry et al. 2001), is presented. Subsequent studies confirmed that this rare allele had the same effect on T1D, psoriasis (PS) (Li et al. 2010), and psoriatic arthritis (PsA) (Budu-Aggrey et al. 2017). On the contrary, this SNP has been associated as a risk factor for the susceptibility of development ankylosing spondylitis (AS) (Ellinghaus et al. 2016), Crohn’s disease (CD) (Ellinghaus et al. 2016; Budu-Aggrey et al. 2017), and ulcerative colitis (UC) (Ellinghaus et al. 2016; Budu-Aggrey et al. 2017).

**Table 1:** rs35667974 frequency in different regional populations (Sherry et al. 2001)

Population	Sample size	Reference allele	Alternative allele
European	175,848	*T =* 0.982274	*C =* 0.017726
African	4974	*T =* 0.9976	*C =* 0.0024
African American	4798	*T =* 0.9975	*C =* 0.0025
Latin American	1776	*T =* 0.9985	*C =* 0.0015
Asian	12,988	*T =* 1.0000	*C =* 0.0000

Chistiakov et al. (2010) have shown that loss-of-function mutations E627X and I923V of MDA5 are associated with lower poly(I:C)–induced interferon-β production in peripheral blood mononuclear cells of type 1 diabetes patients and therefore are T1D protective. It is also stated that in the MDA5 molecule, the I923V amino acid substitution resides in the vicinity to an H927 residue, which contributes to the binding of dsRNA (Yoneyama and Fujita 2008). However, the MDA variant I923V was shown to have a normal ability for binding dsRNA but a 2.5-fold reduced catalytic activity (Shigemoto et al. 2009). Therefore, this polymorphism seems not to affect in a major way the nucleotide acid-binding properties of this cytoplasmic RNA-sensing helicase but alters its function by a still unknown mechanism.

The relation between the *IFHI1* polymorphism and incidence of enterovirus infection in T1D and the association between the MDA5 I923V variant and frequency of enteroviral RNA in T1D patients have been found (Looney et al. 2015). Furthermore, recent studies on MDA5- and MAVS-knockout mice showed a critical role of these proteins in mediating type 1 interferon responses against coxsackie B virus (Wang et al. 2010). A mengovirus leader protein is shown to prevent the expression IFN-β by blocking the dimerization of IRF3 needed for the activation of this factor (Hato et al. 2007). This observation suggests that variants that disrupt IFIH1 function in the host antiviral response have been negatively selected, rather than positively selected because they confer protection from T1D (Crow 2011).

Chow et al. (2018) have extensively analyzed among others the RIG-I-like receptors, while Brisse and Ly (2019) reviewed extensively the evolution and speciation of MDA5 and its related RIG-I. The location of the altered residues in or near the RNA-binding and ATP-binding sites or the filament interface led us to hypothesize that the observed mutations might enhance the stability of the IFIH1 filament by increasing the intrinsic affinity between IFIH1 and dsRNA or between IFIH1 molecules in the filament or by decreasing the efficiency of ATP hydrolysis and, thus, filament disassembly rate (Rice et al. 2014).

This work represents a structural study of the potential role of the shared rs35667974 variant of autoimmune locus *IFIH1*, reported to lead to an inhibited function phenotype encoding an Ile923Val amino acid substitution in the *IFIH1* gene protein product MDA5. The latter is a biologically plausible causal candidate gene shared among several diseases, influencing the control of the local expression of cytokines and chemokines that protect against autoimmunity (Colli et al. 2010). The goal of this work is to explore the unknown still mechanism by which the Ile923Val substitution reduces catalytic activity of human MDA5. In this study we investigated the differences in the interaction of MDA5 with dsRNA between native and the rare variant Ile923Val in the initiation of the inflammation mechanism. Accordingly, we aimed to investigate the dynamic behavior of human MDA5/dsRNA complex in aqueous environment in presence of Ile923 or Val923 when uracil 2′-O is methylated or not. This structural analysis of rare shared genetic susceptibility or protection loci may provide insights to our understanding of the pathophysiology of autoimmune diseases and the research findings may affect the better management of the diseases under study.

## Materials and Methods

### Sequence Retrieval, Phylogenetic Tree Construction and Positive Selection Analysis

Protein sequence of *Homo sapiens* (sequence ID: NP_071451.2) was retrieved from the UNIPROT database (The UniProt Consortium 2021). To find homologs across species, BLAST searches were performed with Mega BLAST (National Center for Biotechnology Information, NCBI, Bethesda, MD, USA) at the RefSeq and NR protein database (and PDB and UniProt) using Blastp (protein–protein BLAST) with default parameters (Altschul et al. 1997). 1000 homologs to the human MDA5 proteins were initially selected and an across species selection focusing on the C-terminal domain containing the sequence around the human I923V substitution was used to identify this variation in other species. Clustal Omega, the multiple sequence alignment program (Clustal-O) (Sievers et al. 2011), and T-Coffee multiple sequence alignment server (Notredame et al. 2000; Di Tommaso et al. 2011) were used to perform protein sequence alignments and Unipro UGENE platform bioinformatics software (Okonechnikov et al. 2012) to selectively visualize multiple alignments. Evolutionary analysis is used to identify positions on the protein sequences that are heavily conserved across species, indicating structural importance (Andreou et al. 2018). The phylogenetic tree was constructed using the Maximum Likelihood method (Nei and Kumar 2000) and Tamura–Nei model (Tamura and Nei 1993) with 500 bootstrap replicates (Felsenstein 1985). Initial tree(s) for the heuristic search were obtained automatically by applying Neighbor-Join and BioNJ algorithms to a matrix of pairwise distances estimated using the Tamura–Nei model and then selecting the topology with superior log-likelihood value. The phylogenetic analysis involved 52 homolog nucleotide sequences (39 orthologs and 13 paralogs) of the human *IFIH1* gene. Codon positions included were 1st + 2nd + 3rd + Noncoding. There were a total of 3729 positions in the final dataset. Evolutionary analyses were conducted using the MEGA11 software package (Tamura et al. 2021). To detect whether the IFIH1 gene has evolved adaptively, we have used the CODEML program in the PAML v4.9j software package (Yang 2007). The nucleotide sequence and corresponding protein sequence alignment file were submitted to PAL2NAL (Suyama et al. 2006) to form the appropriate CODEML input nucleotide alignment files. The positive selection analyses for the ortholog MDA5 genes were performed using site and branch-site models (Yang et al. 2005; Yang and Bielawski 2000). The nonsynonymous/synonymous substitution rate ratio (*ω* = dN/dS) provides a measure of selective pressure at the amino acid level. The magnitude of the value of dN/dS (*ω*) represents the types of selection: *ω* < 1 for negative selection, *ω* = 1 for neutral selection, and *ω* > 1 for positive selection (Yang et al. 2005). In CODEML the site models (M0, M1, M2, M3, M7, and M8) and branch-site models (Clades A and C) were selected to perform the positive selection analysis (Bielawski and Yang 2004; Yang and Nielsen 2002). In the site models, the likelihood ratio test (LRT) was used to test positive selection by comparing the three pairs of models (M0/M3, M2/ M1, and M7/M8). The analysis was performed both for the full-length sequence as well as the C-Terminal Domain (CTD) sequence.

### Structural Analysis and Molecular Dynamics Simulations

The cryoelectron microscopy (cryo-EM) structure of hMDA5–dsRNA filament in the presence of ATP (PDB ID: 6GKM) (Yu et al. 2018) (Berman et al. 2000) was used as model system for the molecular dynamics (MD) simulations. All protein residues resolved (307–1020), the 14 base pairs of double-stranded RNA (dsRNA) and the coordinated zinc were retained, whereas missing residues were modeled using the SWISS-MODEL server (Waterhouse et al. 2018). Force field parameters and hydrogen atoms were added using the XLEaP module of AMBER 18 (Case et al. 2005). The AMBER force fields *ff14SB* (Maier et al. 2015) and *ff99OL3* (Zgarbová et al. 2011) were used for the protein and RNA, respectively, with the *modrna08* (Aduri et al. 2007) parameters for modified nucleosides. The I923V mutation was introduced in MDA5 by manually removing the C^δ^ methyl group of I923, while the modified ribose of U12 was methylated at 2′-O using the *modrna08* library residue MRU. The zinc ion was bonded with the 4 cysteine residues 907, 910, 962, and 964, using appropriate force field parameters to retain a tetrahedral coordination sphere (Zn–S bond lengths of 2.35 Å with 50 kcal·mol^–1^·Å^–2^ force constants and S–Zn–S angles of 109.5 deg with 25 kcal·mol^–1^·rad^–2^).

In this way, we prepared 4 systems for the MD simulations: (i) the native MDA5–dsRNA, (ii) MDA5(V923)–dsRNA, (iii) MDA5–dsRNA(2′OMe), and (iv) MDA5(V923)–dsRNA(2′OMe). All systems were solvated in truncated octahedral solvent boxes of pre-equilibrated TIP3P water molecules, with a minimum buffer of 10 Å around the complex and then the required number of counter ions was added to obtain charge neutralization of the systems. MD simulations were performed with the GPU-accelerated version of PMEMD (Salomon-Ferrer et al. 2013) module in AMBER 18 and a time step of 2 fs. Temperature was regulated using the Langevin thermostat with a collision frequency of 1.0 ps^–1^, while pressure was regulated using the Berendsen barostat with a pressure relaxation time of 1.0 ps. SHAKE was used to constraint bonds involving hydrogen atoms with a tolerance of 10^–6^ Å, whereas non-bonded interactions were calculated with a direct space limit of 10 Å.

Energy minimization was performed initially for 10,000 steps with positional restraints of 100 kcal·mol^–1^·Å^–2^ force constant on the non-hydrogen atoms of MDA5–dsRNA. The solvent was then equilibrated at 300 K and 1 atm through short rounds of simulations in the NVT and NPT ensembles, 100 ps and 400 ps, respectively, while keeping the restrains on non-hydrogen atoms of the solute. Subsequently, energy minimization was performed for 10,000 steps, but with positional restraints of 10 kcal·mol^–1^·Å^–2^ only on the C^α^ atoms of MDA5 and phosphate backbone of the dsRNA. In 3 subsequent rounds of NPT equilibration the positional restraints were gradually relaxed (10.0, 1.0, 0.1 kcal·mol^–1^·Å^–2^) throughout 1 ns, followed by 9 ns of unrestraint equilibration under constant pressure. After these initial 10 ns of equilibration (not used in the analysis), 100 ns of production simulations in the NPT ensemble were performed for each system at 300 K and 1 atm, while storing snapshots of the system every 5.0 ps for analysis using the CPPTRAJ module of AMBER 18 (Roe and Cheatham 2013). All figures depicting 3D models were generated using the PyMOL molecular graphics system (v.2.3 open-source build).

## Results

### Phylogenetic Analysis of the Ile923Val Substitution of MDA5

**Fig. 1:**
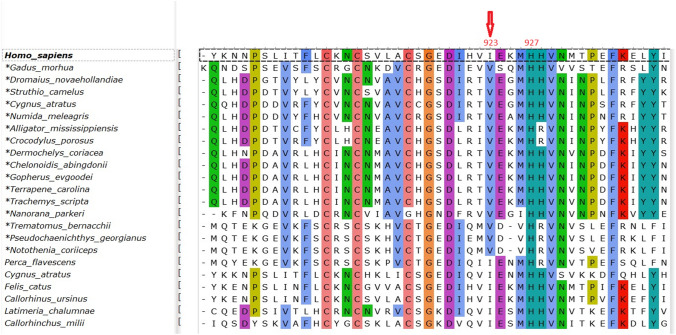
Species sequence alignment around polymorphism of the IFIH1 proteins. SNP under consideration at position 923 in the human protein is indicated by a red arrow. The asterisk (*) before species indicates the existence of a Val amino acid residue at the equivalent position. This figure only illustrates the polymorphism (V923) and not the (I923) sequence conservation. A totally conserved histidine in position 927 is also highlighted

**Fig. 2:**
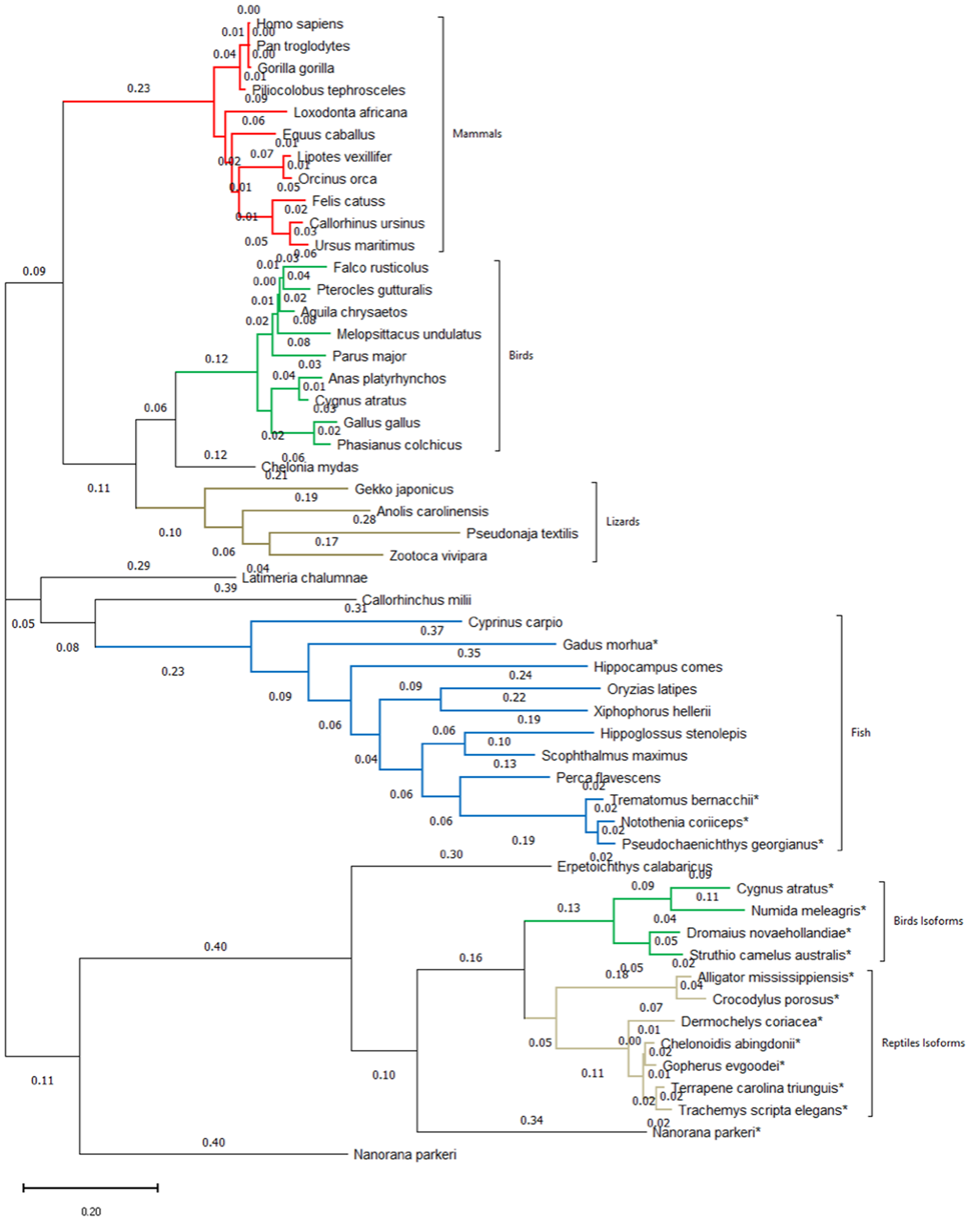
Phylogenetic tree of an across species selection of hMDA5 homologous sequences. The phylogenetic tree is showing the distribution of Ile to Val mutation on the RD/CTD/RS domain of the helicases. Species names marked with asterisk (*) have the Val amino acid occupying the same position

Evolution and speciation of MDA5 and its related RIG-I are reviewed extensively by (Brisse and Ly 2019). Here the *IFIH1* evolution was employed to define conservation elements in the MDA5 sequence in relation to the particular polymorphism. Evolutionary analysis revealed heavy sequence conservation among MDA5 of different species (984 out of 1000 sequences investigated have an isoleucine at the equivalent position of hMDA #923) in the RD/CTD domain, indicating structural/functional importance. The polymorphism rs35667974 in exon 14 of the human gene causes a conserved amino acid mutation at position 923 from Ile to Val in hMDA5. However, there are another sixteen distant species that have the same position occupied by a valine (Fig. 1) indicating the viability of this change across species in the RD/CTD domain of MDA5 and the closely homologous RS/GY domain of isoforms X1, X3, and DHX58 helicases (Fig. 2). In addition, the sequence alignment of the region around the hMDA #923 position (the MDAs CTD interaction loop) reveals a moderate to highly conserved sequence among distant species indicating the across species functional importance of the region.

### Detection of Positive Selection

To detect whether IFIH1 gene has evolved adaptively site models and branch-site models were used to perform positive selection analysis of orthologs of the whole gene and the CTD domain. In the site models, no positive selection sites were identified for the CTD domain (Table 2). M0 implies a constant rate of evolution (*ω* = dN/dS = 0.2) (Table 2). Some sites that had undergone positive selection were identified using the M2- and M8-site models method for the whole gene (Suppl. Table 1), although not in the RNA interacting regions and the CTD domain. In the site models, ω (dN/dS) is < 1 which indicates a highly conserved gene (Table 2, Suppl. Table 1). In the branch-site model the human branch (as well as the primate’s branch) used as the foreground clade, the ω value was low, and no sites with posterior probability greater than 0.85 were identified (Suppl. Table 2).

**Table 2:** Test for positive selection among codons of CTD domain orthologs using site models

Model	Np	Estimates of parameters	InL	Mondel comparison	2ΔInL	Positively selected sites
M0	77	*ω* = 0.20640	− 3710.521608	M3 vs M0	232.174724(M3 vs M0)	None
M3	81	*p*: 0.33141 0.55271 0.11588	− 3594.434246			None
		*ω*: 0.03587 0.24706 0.89173				
M1	78	*p*: 0.77042 0.22958	− 3641.383413	M2 vs M1	0(M2 vsM1)	None
		*ω*: 0.15817 1.00000				
M2	80	*p*: 0.77042 0.14282 0.08675	− 3641.383413			5H
		*ω*: 0.15817 1.00000 1.00000				
M7	78	*p* = 0.68777 *q* = 1.97666	− 3594.244452	M8 vs M7	7.698494(M8 vsM7)	None
M8	80	*p*_*0*_ = 0.94718 *p* = 0.88880 *q* = 3.43223	− 3590.395205			5H
		(*p*_*1*_ = 0.05282) *ω* = 1.22219				

*Np* number of estimated parameters, *lnL* log-likelihood score, *2ΔInL* twice the log-likelihood difference of the model compared, Positive selection sites are inferred at posterior probabilities > 95% shown in bold asterisk (*); with those reaching > 99% shown in two bold asterisks (**)

All amino acids are located on the reference sequence 1st sequence: 1.Homo_ sapiens, based on the mul

In particular, for the branch-site model C for the CTD domain (Suppl. Table 3), 33% of the sites are evolving at category *ω*_0_ = 0. 036. As sites evolving under this category do not distinguish between branch types, both branch types have the same value of ω for sites under this category. Moreover, 55% of the sites are evolving under category ω_2_. Yet, these have ω values that are conditional on the branch type (*ω*_20_ = 0.25 and *ω*_21_ = 0). Also, for the branch-site model C for the 39 ortholog MDA5 genes (Suppl. Table 4), 33% of the sites are evolving at category *ω*_0_ = 0. 027. On the other hand, 41% of the sites are evolving under category ω_2_. Yet, these have ω values that are conditional on the branch type (*ω*_20_ = 0.25 and *ω*_21_ = 15.32).

### Structural Analysis

**Fig. 3:**
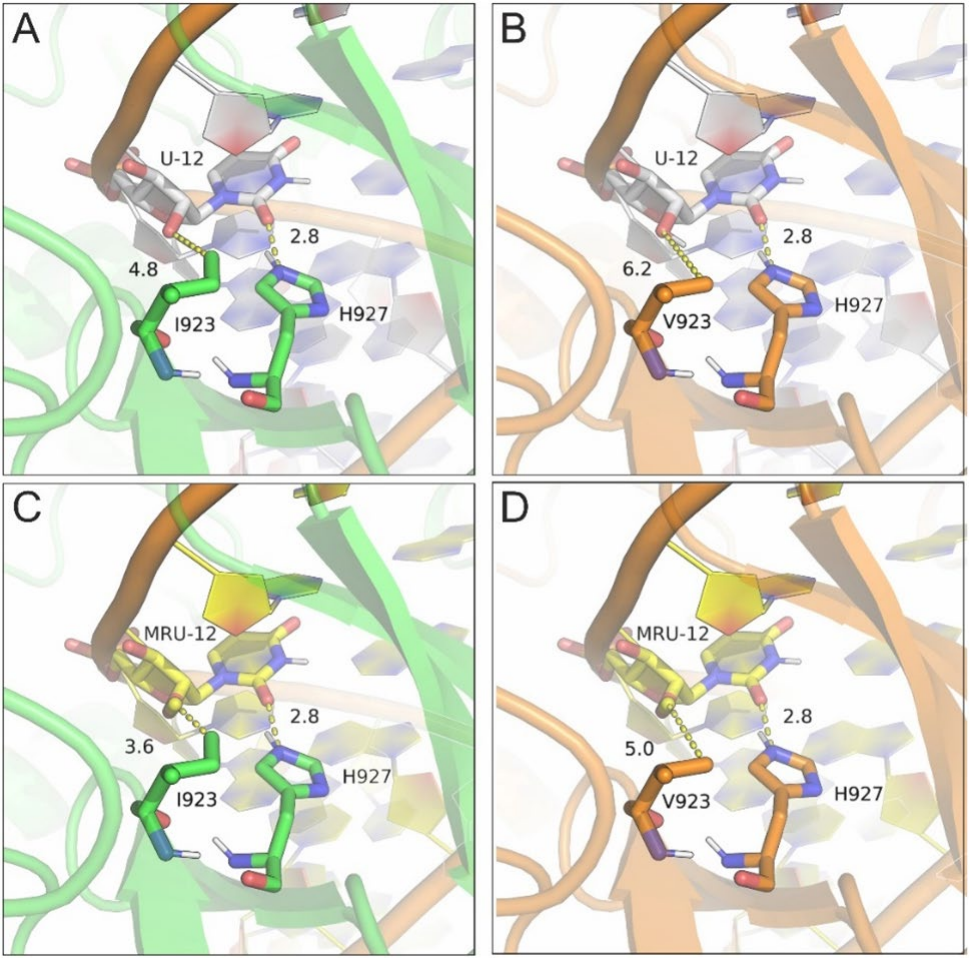
Close-up view of the MDA5/dsRNA contact interface for all distinct cases. **A** Native contacts of MDA5/dsRNA from the CryoEM structure of the complex (PDB ID: 6GKM) illustrating the hydrogen bond between H927 and U12 and the closest distance (in Å) between I923 and 2΄-OH of U12. (B) Model of I923V mutant indicating the longer distance between V923 and U12. **C** Model of native MDA5 interacting with 2΄-*O*-methylated U12 (designated as MRU-12). **D** Model of I923V MDA5 with 2-O’-methylated U12 (MRU-12)

The conducted evolutionary analysis demonstrates that the Ile923Val substitution is not a unique variant in the human species, since Val exists in the MDA5 sequence position in other species too. MDA5 is a viral double-stranded RNA (dsRNA) receptor that plays a key role in antiviral immunity through its distinct specificity for viral RNA (Wu et al. 2013). It has been shown that 2′-O-methylation of viral mRNA is important for the innate immune responses, therefore it has been suggested that 2′-O-methylation is a molecular signature for the distinction of self- versus non-self mRNA. (Zust et al. 2011). To investigate the potential role of Ile923Val substitution in the missense IFIH1 variant rs35667974, we analyzed the cryoelectron microscopy (cryo-EM) structure of MDA5–dsRNA filament in the presence of ATP (PDB ID: 6GKM) (Yu et al. 2018). Position 923 is located on the loop 921–927 that interacts directly with the dsRNA (Fig. 3A). In particular, Ile923 is located 4.8 Å from 2′-ΟΗ of uridine U12 and their interaction is stabilized through a hydrogen bond between the adjacent His927 and the uracil base. Substitution of Ile923 by Val in rs35667974 variant is not expected to introduce any steric clashes, rather than minimize interactions with 2′-ΟΗ of uridine U12 (Fig. 3B). In case RNA is methylated at the ribose of U12, then the natural MDA5 variant with Ile923 displays a favorable van der Waals contact with 2′-OMe group of U12 at 3.6 Å (Fig. 3C), whereas Val923 of the rs35667974 variant is located at 5.0 Å (Fig. 3D). These differences cannot suggest a major effect of the Ile923Val substitution per se; however, subtle structural changes often lead to significant functional changes through disruption of the structural dynamics of the system.

### Molecular Dynamics Calculations

**Fig. 4:**
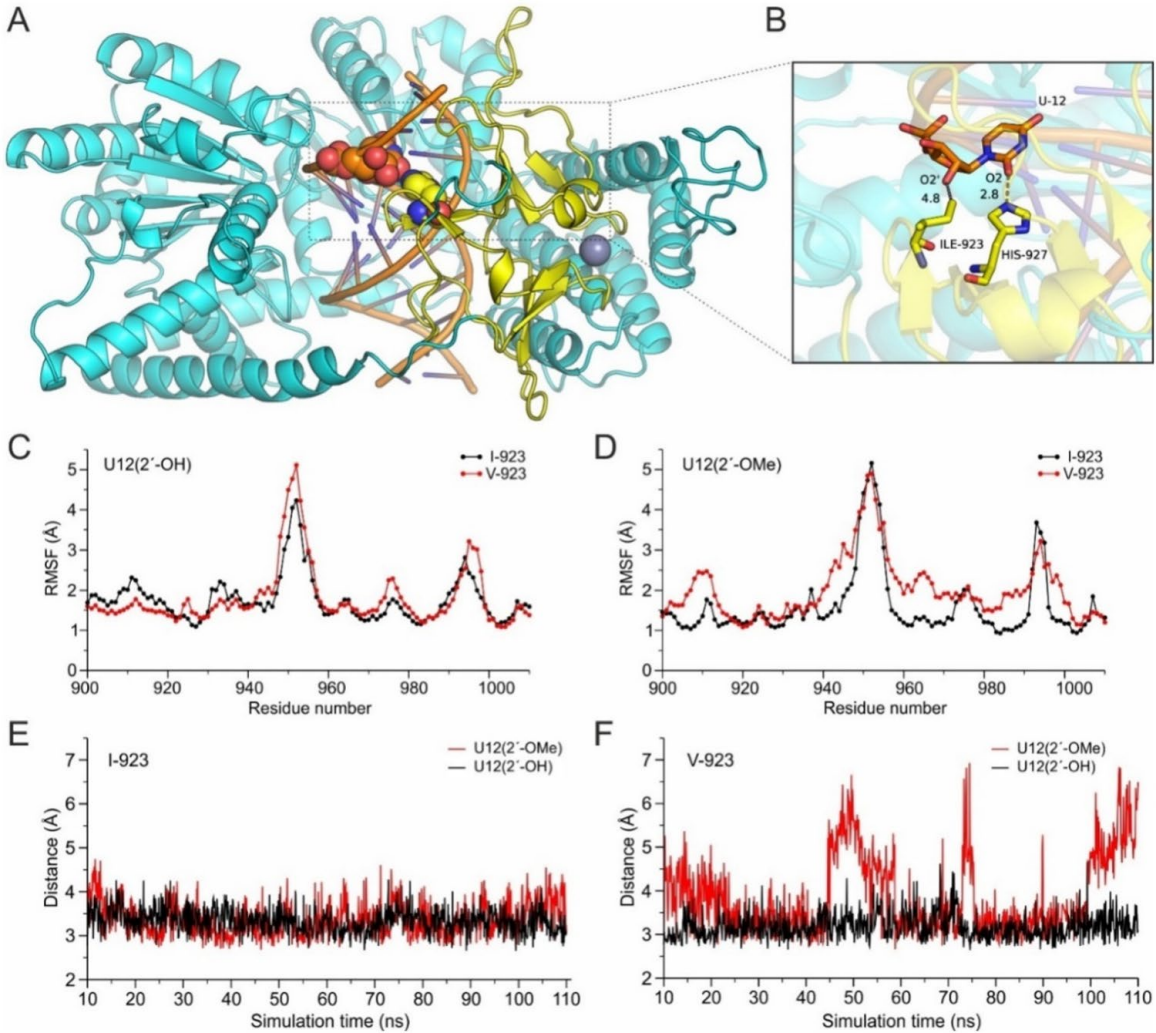
Molecular Dynamics (MDs) simulation results. **A** Cartoon representation of the CryoEM structure of the complex between MDA5 (cyan) and dsRNA (orange), illustrating the cooperative assembly of MDA5 into helical filaments on dsRNA (PDB ID: 6GKM). The carboxy-terminal region of MDA5 is highlighted in yellow (residues 900–1010) and the magenta sphere is a Zn(II) ion coordinated by 4 cysteine residues. **B** Close-up view of a key hydrogen-bonding interaction between His927 (yellow C atoms) and U12 of the dsRNA (orange C atoms), indicating the position of Ile923 and its distance from the 2′-O atom of U12. **C** Root-mean-square atomic fluctuations (RMSF) of the Cα atoms in the region 900–1010 of MDA5 (highlighted with yellow in A), which were extracted from the simulations of MDA5–dsRNA complex for the native and the MDA5 bearing the I923V mutation. **D** Same as in (**C**), but extracted from the simulations of the native and the MDA5–dsRNA bearing the I923V mutation with methylated ribose of U12. **E** Hydrogen-bonding distance between His927(Nε2) and U12(O2) atoms as a function of simulation time of the native MDA5–dsRNA for the native MDA5 with the unmethylated RNA (2′-ΟΗ, black) and the U12 ribose methylated (2′-ΟMe, red). (F) Same as in (E), but from the simulations of MDA5–dsRNA of the mutant MDA5 bearing the I923V mutation with the unmethylated RNA (2´-ΟΗ, black) and the U12 ribose methylated (2′-ΟMe, red)

With the aim to investigate the effect of I923V mutation of MDA5 in its interaction with dsRNA, both native and 2′-*Ο*-methylated, we have employed molecular dynamics simulations of 4 systems at the 100-ns timescale. The dynamics of the systems were monitored using the root-mean-square fluctuations (RMSF) of each protein residue and the hydrogen-bonding distance of H927 with U12 (Fig. 4). Our calculations suggest that mutation of I923 to V923 in MDA5 resulted in minor perturbation of the dynamics within the RNA contact region (residues 923–934) and the interprotein interaction loop (950–955) of the complex with native dsRNA (Fig. 4C). This observation was similar in the case of 2′-*O*-methylation in U12, although a more pronounced effect was observed in the overall dynamics of the carboxy-terminal region of MDA5 V923 mutant (Fig. 4D). Now considering the key hydrogen-bonding interaction of the adjacent H927 residue with the uracil base, our MD simulations suggest that methylation at 2′-*O* does not affect it in the native MDA5 (Fig. 4E). However, the V923 mutation does not affect the hydrogen bond of H927 in the native dsRNA but displayed a destabilizing effect in the presence of 2′-*O*-methylation (Fig. 4F). Taken together, our MD simulations suggest that although the effect of I923V mutation of MDA5 in the interaction with native dsRNA is marginal, its effect in the dynamics and stability of the MDA5/RNA complex is more significant when uracil is 2′-*O*-methylated.

## Discussion

This study represents an evolutionary and structural investigation of the role of the shared rs35667974 variant of autoimmune locus *IFIH1*, reported to lead to a modified functionality phenotype for MDA5 (Downes et al. 2010). The application of evolutionary site and branch-site models to perform positive selection analysis indicates no positive selection sites in the RNA interaction sites where the rs35667974 polymorphism resides. Neither the branch-site models indicate any particular sites undergone positive selection in the CTD domain. This is in agreement with the very low appearance frequencies of the Ile923Val variant even among the human population (Table 1). Nevertheless, the European population in comparison with the others has a difference in the frequency of the C allele of one order of magnitude. Without overlooking the small sample size, the geography distribution of the polymorphism under study shows that its appearance in the European population as the starting point maybe due to living conditions and nutrition that have changed in recent years. The structural investigation of the role of the examined SNP was performed by examining the structure of the dsRNA–MDA5 (native and mutant) complex. At the dsRNA–MDA5 complex formation level the introduction of the Ile923Val mutation influences the interaction of the protein C-terminal domain (CTD) with the dsRNA with the introduction of a hydrophobic cavity next to the ribose sugar of the one strand of the dsRNA. We have shown that in the case of methylated RNA at the phosphoribose chain of the one strand further dynamic effects may influence the interaction of the mutant while not affecting the wild type. This is in agreement with experimental studies (Looney et al. 2015; Brisse and Ly 2019) showing that the effect of the Ile923Val polymorphism identified in the vicinity of an MDA5–dsDNA interaction point may not influence the native dsRNA-binding properties but alter by 2.5-fold reduction the catalytic activity (Shigemoto et al. 2009). Also the molecular dynamic studies have shown the critical role of the methylation in the mutant concerning the mobility and stability of MDA5 loops 941–959 and 970–977 involved in dsRNA interaction and interprotein interaction in the MDA filament formation. Such effects may hinder the MDA5 filament assembly, the MDA5–MAVS association, and the MAVS filament assembly that further activate the expression of the type I interferon genes (IFN1: IFNα and IFNβ). In the cases of T1D and PsA, as a consequence, reduced levels of MDA5 protein activity and therefore lower IFNβ production protects against autoimmunity. These observations suggest that several IFIH1 variants, predicted to affect the interaction of MDA5 to MAVS and reduction of IFNβ production would decrease the risk of diseases, while normal MDA5 function is associated with them (Shigemoto et al. 2009). This leads to the conclusion that as in the case of viral RNA, self-dsRNA methylation, an RNA epigenetic effect, becomes through the introduction of the identified mutation, an important selection/activation factor for the introduction of protective effect in some autoimmune diseases. The results from the present study expand the knowledge of the biological significance of rs35667974 SNP of *IFIH1* locus in the development of the aforementioned diseases and highlight the importance of studies of shared genes by multiple autoimmune diseases. However, in population studies for the genetic association of SNPs with autoimmune diseases, using, e.g., PCR-RFLPs, sequencing, or genotyping chips, a case of RNA methylation is not taken into account. It is important therefore to know the state of methylation of the interacting dsRNA and its effect on the allele MDA5. As in the case of the viral dsRNA recognition (Wu et al. 2013) and the distinction between self- and non-self mRNA (Züst et al. 2011), the loss of a methyl group from the interacting MDA5, as in the case of the mutant Ile923Val MDA5, may affect the filament formation and the induction of type I interferon. Of note, Plenge et al. (2013) had previously discussed the potential of a rare variant in a causal disease gene to represent a putative therapeutic target for pharmaceutical intervention. To this end, the biological function of the causative variant has to be known in any attempt to link genetic findings with a new therapeutic target. Therefore, locating the position of a causative variant in the 3D structure of the respective protein and investigating its role from the structural/functional viewpoint in a pathogenetic pathway leading to an autoimmune disease seem to be of pivotal importance for the further management and a better treatment of the patients. There remains a supreme need to move beyond discovery of associated SNPs to a deeper understanding of causative variants to elucidate the molecular mechanisms and pathways of disease. Further structural functional analysis is required to investigate the binding of ribose-2′-*Ο*-methylated self-dsRNA to MDA5 and how this affects MDA5 fibril assembly. The elusive biological role for 2′-*Ο*-methylated of mRNA as a sensor of distinction between viral and self-mRNA in the induction of type I interferon may have been extended to a protective sensor in certain autoimmunopathies.

## Conclusion

The rare rs35667974 *IFIH1* gene polymorphism protects from T1D, PS, and PsA, while the *IFIH1* allele carried by the majority of the population predispose to the diseases. The finding that the mutant Ile923Val MDA5 functions differently in the interaction with self-dsRNA and in particular with 2′-*O*-methylated one suggests that in some cases variants interacting with methylated dsRNA disrupt MDA5 filament formation, MAVS interaction and filament formation, and IFN signaling, as in the host antiviral response, may possible have been negatively selected, because they confer protection from diseases.

## Supplementary Information

Below is the link to the electronic supplementary material.

Supplementary file1 (DOCX 24 kb)

## Data Availability

The datasets used and/or analyzed during the current study are available from the corresponding author on request.
