# Supplementary material for: Is the Association of the Rare rs35667974 IFIH1 Gene Polymorphism With Autoimmune Diseases a Case of RNA Epigenetics?
Source: J Mol Evol. 2023 Jan 18;91(2):204–13. doi: 10.1007/s00239-022-10090-0 (PMC10082101; doi:10.1007/s00239-022-10090-0)
Supplement: Supplementary file 1 — Supplementary file1 (DOCX 24 kb) [file 239_2022_10090_MOESM1_ESM.docx]

**Supplementary Material**

**Is the association of the rare rs35667974 *IFIH1* gene polymorphism with autoimmune diseases a case of RNA epigenetics?**

**Athena Andreou^1^, Athanasios Papakyriakou^2^, Maria I. Zervou^3^, George N. Goulielmos^3,4^, Elias E. Eliopoulos^1*^**

1 Laboratory of Genetics, Department of Biotechnology, Agricultural University of Athens, 11855 Athens;

2 Institute of Biosciences and Applications, National Centre for Scientific Research "Demokritos", 15341, Athens, Greece;

3 Section of Molecular Pathology and Human Genetics, Department of Internal Medicine, School of Medicine, University of Crete, 71003 Heraklion;

4 Department of Internal Medicine, University Hospital of Heraklion, 71500 Heraklion, Greece.

*** Correspondence:** eliop@aua.gr; Tel.: +30 210 529 4223

Orcid: AA, 0000-0003-4683-8071; AP, 0000-0003-3931-6232; MIZ, 0000-0001-6750-3452; EEE, 0000-0002-0919-0571.

| **Model** | **Np** | **Estimates of parameters** | **InL** | **Mondel Comparison** | **2ΔInL** | **Positively selected sites** |
| --- | --- | --- | --- | --- | --- | --- |
| M0 | 77 | ω= 0.25631 | -58365.482417 | M3 vs M0 | 4794.654814 (M3 vs M0) | none |
| M3 | 81 | p: 0.32717 0.40807 0.26477  ω: 0.02722 0.25219 0.85659 | -55968.155010 |  |  | none |
| M1 | 78 | p: 0.60660 0.39340  ω: 0.12395 1.00000 | -56522.109823 | M2 vs M1 | 40.842683( M2 vs M1) | none |
| M2 | 80 | p: 0.59753 0.37006 0.03241  ω: 0.12571 1.00000 2.19023 | -56501.686889 |  |  | 14 T, **17 N****, **47 A***, 82 L, 93 R,**98 P***,  122E, 131 Q, 155 I,**162 A****, 247 D,  272 V ,288 S, 348 M, 413 V, 501 W, 659 C , 997 L,**1018 H*** |
| M7 | 78 | p = 0.45682 q = 0.93125 | -55915.234534 | M8 vs M7 | 62.478956(M8 vs M7) | none |
| M8 | 80 | p0 = 0.91358 p = 0.54507 q = 1.51458  (p1 = 0.08642) ω = 1.31232 | -55883.995056 |  |  | 14 T, **17 N*,** 27 A ,**47 A*,** 58 A, 63 M,71 S, 81 H,82 L,93 R,94 R**, 98 P***,122 E, 131 Q,145 D , 155 I,158 R,**162 A***,238 E ,247 D ,248 C ,257 N , 272 V,281 Q , 288 S,338 E, 339 V,346 W, 348 M,353 S , 375Α, 413 V, 501 W, 648 L, 649 D, 659 C , 686 Τ, 997 L , 1016 Α,**1018 H*** |

**Suppl. Table 1** Test for positive selection among codons of the MDA5 orthologs genes using site models.

Positive selection analyses are performed by using ‘Codeml’ implemented in PAML. Np: number of estimated parameters; lnL: log likelihood score;2ΔInL : twice the log-likelihood difference of the two compared model; Positive- selection sites are inferred at posterior probabilities >95% shown in bold asterisk (*); with those reaching >99% shown in two bold asterisks (**).

Note: All amino acids are located on the reference sequence 1^st^ sequence : 1.Homo_ sapiens, based on the multiple alignment results.

**Suppl. Table 2** Parameters estimation and likelihood ratio tests for the branch-site model A for the 39 orthologs *IFIH1* genes

| site class | proportion | background  ω | foreground  ω | Positively selected sites |
| --- | --- | --- | --- | --- |
| 0  1  2a  2b | 0.08745 0.08338 0.42446 0.40471 | 0.07209  0.56315  0.07209  0.56315 | 0.07209 0.56315 0.00000 0.00000 | none |

Note: Positive-selection site are inferred at poster probabilities > 80%

**Suppl. Table 3** Parameters estimation for the branch-site model C for the CTD domain.

| site class | 0 | 1 | 2 |
| --- | --- | --- | --- |
| proportion | 0.33131 | 0.11545 | 0.55324 |
| branch type 0: | 0.03588 | 0.89359 | 0.24769 |
| branch type 1: | 0.03588 | 0.89359 | 0.00000 |

Note: the columns indicate the ω categories (0, 1, and 2), and the rows below proportion indicate the ω values for the distinct branches. ω0 and ω1 have the same values independently of the branch type, meanwhile ω2 has values that vary across the different branch types

**Suppl. Table 4** Parameters estimation for the branch-site model C for the 39 orthologs MDA5 genes.

| site class | 0 | 1 | 2 |
| --- | --- | --- | --- |
| proportion | 0.32717 | 0.26477 | 0.40807 |
| branch type 0: | 0.02722 | 0.85659 | 0.25219 |
| branch type 1: | 0.02722 | 0.85659 | 15.32525 |

Note: the columns indicate the ω categories (0, 1, and 2), and the rows below proportion indicate the ω values for the distinct branches. ω0 and ω1 have the same values independently of the branch type, while ω2 has values that vary across the different branch types
